# Supplementary material for: Multidimensional poverty reduction effect of climate smart agriculture practices among rural households in Siltie Zone, Central Ethiopia
Source: PLoS One. 2026 May 4;21(5):e0348583. doi: 10.1371/journal.pone.0348583 (PMC13138676; doi:10.1371/journal.pone.0348583)
Supplement: Attachment — Submitted filename: PONE-D-25-39956 (4).pdf [file pone.0348583.s002.pdf]

PLOS ONE

Multidimensional Poverty Reduction Effect of Climate-Smart Agriculture Practices among rural households in Siltie Zone, Central Ethiopia

--Manuscript Draft--

**Manuscript Number:**	PONE-D-25-39956
**Article Type:**	Research Article
**Full Title:**	Multidimensional Poverty Reduction Effect of Climate-Smart Agriculture Practices among rural households in Siltie Zone, Central Ethiopia
**Short Title:**	Multidimensional Poverty Reduction Effect of Climate-Smart Agriculture Practices
**Corresponding Author:**	jemil Yasin shifa, PhD student<br>Addis Ababa University<br>Werabe, ETHIOPIA
**Keywords:**	MPI, CSA, GSEM, PSM, 2SLS, Central Ethiopia
**Abstract:**	This study investigates the effect of Climate-Smart Agricultu[illegible]doption on multidimensional poverty reduction in the Siltie Zone, Centra[illegible]hiopia. Despite growing attention to CSA as a pathway toward sustainable development and poverty reduction, limited empirical evidence exists about its effect on multidimensional poverty. Using cross-sectional data collected from 416 smallholder farmers selected employing multi stage sampling procedure and GSEM, 2SLS and PSM analytical approaches by address potential endogeneity between CSA adoption and MPI. The multidimensional poverty index was constructed to capture deprivations across different dimensions while CSA adoption was measured based on the adoption and intensity of practices implemented. Results reveal that CSA adoption significantly reduces multidimensional poverty. These findings underscore the transformative potential of CSA in enhancing rural multidimensional poverty and highlight the importance of targeted policy interventions that promote different CSA practices adoption as a strategy. The study contributes to the growing body of literature linking sustainable CSA practices to inclusive and multidimensional poverty reduction as development outcomes.
**Order of Authors:**	jemil Yasin shifa, PhD student
	Abrham Seyoum Tsehay, Associate professor
**Additional Information:**	
**Question**	**Response**
**Financial Disclosure**<br><br>Enter a financial disclosure statement that describes the sources of funding for the work included in this submission. Review the submission guidelines for detailed requirements. View published research articles from *PLOS ONE* for specific examples.<br><br>This statement is required for submission and **will appear in the published article** if the submission is accepted. Please make sure it is accurate.	The author(s) received no specific funding for this work.

Powered by Editorial Manager® and ProduXion Manager® from Aries Systems Corporation

**Funded studies**<br>Enter a statement with the following details:<br>• Initials of the authors who received each award<br>• Grant numbers awarded to each author<br>• The full name of each funder<br>• URL of each funder website<br>• Did the sponsors or funders play any role in the study design, data collection and analysis, decision to publish, or preparation of the manuscript?<br><br>Did you receive funding for this work?	
**Competing Interests**<br><br>Use the instructions below to enter a competing interest statement for this submission. On behalf of all authors, disclose any competing interests that could be perceived to bias this work—acknowledging all financial support and any other relevant financial or non-financial competing interests.<br><br>This statement is required for submission and **will appear in the published article** if the submission is accepted. Please make sure it is accurate and that any funding sources listed in your Funding Information later in the submission form are also declared in your Financial Disclosure statement.<br><br>View published research articles from *PLOS ONE* for specific examples.	The authors have declared that no competing interests exist.<br>Addis Ababa University Institutional Review Board (IRB), reference number: number:075/04/2024.

**NO authors have competing interests**<br><br>Enter: *The authors have declared that no competing interests exist.*<br><br>**Authors with competing interests**<br><br>Enter competing interest details beginning with this statement:<br><br>*I have read the journal's policy and the authors of this manuscript have the following competing interests: [insert competing interests here]*<br><br>* typeset	
**Ethics Statement**<br><br>Enter an ethics statement for this submission. This statement is required if the study involved:<br><br>• Human participants<br>• Human specimens or tissue<br>• Vertebrate animals or cephalopods<br>• Vertebrate embryos or tissues<br>• Field research<br><br>Write "N/A" if the submission does not require an ethics statement.<br><br>General guidance is provided below. Consult the submission guidelines for detailed instructions. **Make sure that all information entered here is included in the Methods section of the manuscript.**	This study passed through Addis Ababa University's institutional review board (IRB). The IRB rigorously examined the proposal’s content, its associated research tools, and the informed consent of the respondents. Oral participant consent (since most respondents are illiterate farmers) and an ethical clearance letter are issued ahead of the data collection. The approval no. of this ethical clearance is 075/04/2024.

Powered by Editorial Manager® and ProduXion Manager® from Aries Systems Corporation

**Format for specific study types**<br><br>**Human Subject Research (involving human participants and/or tissue)**<br>• Give the name of the institutional review board or ethics committee that approved the study<br>• Include the approval number and/or a statement indicating approval of this research<br>• Indicate the form of consent obtained (written/oral) or the reason that consent was not obtained (e.g. the data were analyzed anonymously)<br><br>**Animal Research (involving vertebrate animals, embryos or tissues)**<br>• Provide the name of the Institutional Animal Care and Use Committee (IACUC) or other relevant ethics board that reviewed the study protocol, and indicate whether they approved this research or granted a formal waiver of ethical approval<br>• Include an approval number if one was obtained<br>• If the study involved *non-human primates*, add *additional details* about animal welfare and steps taken to ameliorate suffering<br>• If anesthesia, euthanasia, or any kind of animal sacrifice is part of the study, include briefly which substances and/or methods were applied<br><br>**Field Research**<br><br>Include the following details if this study involves the collection of plant, animal, or other materials from a natural setting:<br>• Field permit number<br>• Name of the institution or relevant body that granted permission	
**Data Availability**<br><br>Authors are required to make all data underlying the findings described fully available, without restriction, and from the time of publication. PLOS allows rare exceptions to address legal and ethical concerns. See the PLOS Data Policy and FAQ for detailed information.	No - some restrictions will apply

Powered by Editorial Manager® and ProduXion Manager® from Aries Systems Corporation

A Data Availability Statement describing where the data can be found is required at submission. Your answers to this question constitute the Data Availability Statement and **will be published in the article**, if accepted.<br><br>**Important:** Stating 'data available on request from the author' is not sufficient. If your data are only available upon request, select 'No' for the first question and explain your exceptional situation in the text box.<br><br>Do the authors confirm that all data underlying the findings described in their manuscript are fully available without restriction?	
**Describe where the data may be found in full sentences. If you are copying our sample text, replace any instances of XXX with the appropriate details.**<br><br>• If the data are **held or will be held in a public repository**, include URLs, accession numbers or DOIs. If this information will only be available after acceptance, indicate this by ticking the box below. For example: *All XXX files are available from the XXX database (accession number(s) XXX, XXX.).*<br>• If the data are all contained **within the manuscript and/or Supporting Information files**, enter the following: *All relevant data are within the manuscript and its Supporting Information files.*<br>• If neither of these applies but you are able to provide **details of access elsewhere**, with or without limitations, please do so. For example:<br><br>*Data cannot be shared publicly because of [XXX]. Data are available from the XXX Institutional Data Access / Ethics Committee (contact via XXX) for researchers who meet the criteria for access to confidential data.*<br><br>*The data underlying the results presented in the study are available from (include the name of the third party*	The data used for this study will be provided upon reasonable request from the corresponding author.

and contact information or URL).<br>• This text is appropriate if the data are owned by a third party and authors do not have permission to share the data.<br><br>* typeset	
Additional data availability information:	

Powered by Editorial Manager® and ProduXion Manager® from Aries Systems Corporation

Manuscript

Click here to access/download;Manuscript;Manuscript_anonymous (effect of

Multidimensional Poverty Reduction Effect of Climate Smart Agriculture Practices among rural households in Siltie Zone, Central Ethiopia

Jemil Shifa (PhD Candidate)[a,b*] Abrham Tsehay (PhD, Associate Professor)[b]

[a]Center for Rural Development, Addis Ababa University, Ethiopia

[a]Rural development, Werabe university, Ethiopia; [b]Rural Development, Addis Ababa university, Addis Ababa, Ethiopia

*Corresponding author (Jemil Yasin Shifa)

Email: yasin.jemil73@gmail.com/jemilyasin@wru.edu.et

Telephone: 0912684698

Multidimensional Poverty Reduction Effect of Climate Smart Agriculture Practices among rural households in Siltie Zone, Central Ethiopia

ABSTRACT

This study investigates the effect of Climate-Smart Agriculture adoption on multidimensional poverty reduction in the Siltie Zone, Central Ethiopia. Despite growing attention to CSA as a pathway toward sustainable development and poverty reduction, limited empirical evidence exists about its effect on multidimensional poverty. Using cross-sectional data collected from 416 smallholder farmers selected employing multi stage sampling procedure and GSEM, 2SLS and PSM analytical approaches by address potential endogeneity between CSA adoption and MPI. The multidimensional poverty index was constructed to capture deprivations across different dimensions while CSA adoption was measured based on the adoption and intensity of practices implemented. Results reveal that CSA adoption significantly reduces multidimensional poverty. These findings underscore the transformative potential of CSA in enhancing rural multidimensional poverty and highlight the importance of targeted policy interventions that promote different CSA practices adoption as a strategy. The study contributes to the growing body of literature linking sustainable CSA practices to inclusive and multidimensional poverty reduction as development outcomes.

KEYWORDS: MPI, CSA, GSEM, PSM, 2SLS, Central Ethiopia

1. Introduction

Agriculture remains the backbone of Ethiopia's economy and contributes significantly to both the national GDP and rural livelihoods with over 70% of the workforce employed, however, the sector is increasingly threatened by climate change, erratic rainfall, and land degradation, which disproportionately affect smallholder farmers and keep them in a state of multifaceted poverty. Climate-Smart Agriculture (CSA) has emerged as a viable approach to address these problems (Teklu, Simane, Bezabih, et al., 2022; Zerssa et al., 2021)

Varies studies supports the contribution of CSA technologies for the improvement of food security and agricultural productivity however, little is known about how it impacts multifaceted poverty in general and especially in developing nations like Ethiopia where poverty is pervasive across different locations and agroecological zones (Adimassu et al., 2025; Ali et al., 2023; Teklu, Simane, & Bezabih, 2022)

A numbers of previous studies focused on Food security effects of CSA adoption, but this study analyze MPI effects of different CSA practices adoption. Furthermore, this study addressed the potential endogeneity between CSA adoption and poverty outcomes through employing different analytical models including GSEM, PSM and 2SLS method of analysis. This study aims to fill these gaps by examining the causal relationship between CSA adoption and multidimensional poverty reduction in the study area using a cross-sectional survey. Therefore, this study provides evidence for policy makers and practitioners to create target climate smart agriculture technologies scale up and equitable interventions across different locations.

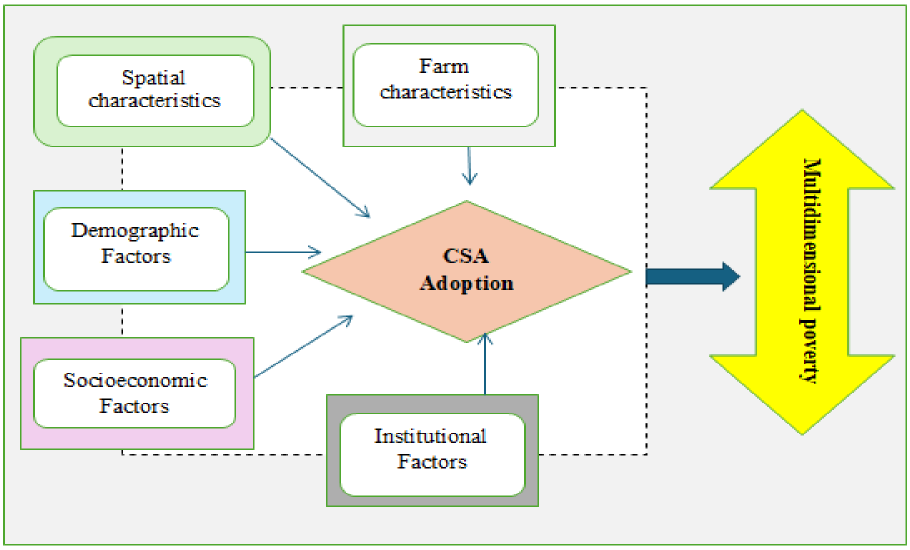

Figure 1: Conceptual framework of the study. Source: based on literature review (2024)

2. Literature review

2.1. Theoretical literature

It is necessary to establish theoretical frameworks in order to comprehend the connection between the adoption of CSA technologies and poverty reduction.

2.1.1. Framework for Sustainable Livelihoods (SLF)

Sustainable Livelihoods Framework (SLF), which offers a comprehensive lens that, shows the way rural households use various resources/ livelihoods, vulnerability contexts, policies institutions and procedures, livelihood strategies and livelihood outcomes (DFID, 1997). CSA technologies as one of strategies households used to respond to climate change shocks and maintain their economic and social wellbeing since it increases resilience to climate-related risks influencing productivity and adaptability (Adimassu et al., 2025; Teklu, Simane, Bezabih, et al., 2022)

2.1.2. Technology Adoption Theories

The Diffusion of Innovations Theory (Rogers, 1962) suggests that adoption is a social process influenced by the perceived attributes of the technology, communication channels,

and social system. Furthermore, economic constraints model states that adoption decisions are influenced by factors such as income, credit access, and other factors.

2.1.3. Capability Approach and Multidimensional Poverty

The Capability Approach, developed (Sen, 1976), shifts the focus of poverty analysis from income to capabilities. The model states that individuals have to live the life they value and measures poverty in different dimensions including health, education, and living standards. Thus CSA practices can be a vehicle to increase productivity and adaptation to climate shocks consequently leading to reducing poverty.

2.2. Empirical review

2.2.1. CSA and Poverty Reduction

Empirical studies highlighted that CSA has role in enhancing agricultural productivity, resilience, and rural livelihoods (Adimassu et al., 2025; Ali et al., 2023; Nkumulwa & Pauline, 2021) However, fewe studies have examined its effect on multidimensional poverty, particularly in consideration with agroecological and spatial variations. For instance, (Ali et al., 2023) found that Farmers who adopt CSA in the Ethiopian Rift Valley enjoyed higher food consumption scores and lower multidimensional poverty. Similarly, (Tesfaye et al., 2021) Uses panel data from Ethiopia to analyse conservation agriculture techniques (minimum tillage, cereal-legume intercropping) finds that these practices significantly reduce the incidence and depth of poverty, especially in rainfall-stressed areas.

2.2.2. Methodological Approaches

Previous studies employed different methodological approach to investigate the effects of CSA technologies adoption on food security/welfare/poverty. For instance, endogenous switch regression used by (Ali et al., 2023; Tesfaye et al., 2021) propensity score matching (PSM) used by (Belay et al., 2023; Midjangninou et al., 2024; Radeny et al., 2022)

Difference-in-Differences used by (Samuel et al., 2024). Nevertheless, each of them has advantages and limitations a growing number of studies acknowledge the endogeneity problem. Though, limited studies uses 2SLS (control endogeneity problem by using appropriate instrument variables) (Bollen, 1996) and GSEM (appropriate for latent and mediation variables and accommodates binary, ordinal and count dependent variable) (Muthén, 1984). This study simultaneously employed GSEM, PSM and 2SLS analytical models to examine the impact of multiple CSA practices adoption on multidimensional poverty**.**

3. Methodology of the study

3.1. Description of the study area

This study was conducted in the Central Ethiopia Region, Silte Zone, which is in the South of Ethiopia and encompasses nine districts. The eco-region lies between 7° 49' 56" N latitude to 38° 16' 7" E longitude, with an elevation of 1967 meters. In the zone, there are two main livelihoods: Enset with mixed crop production in the highland and mixed crop production in the midland.

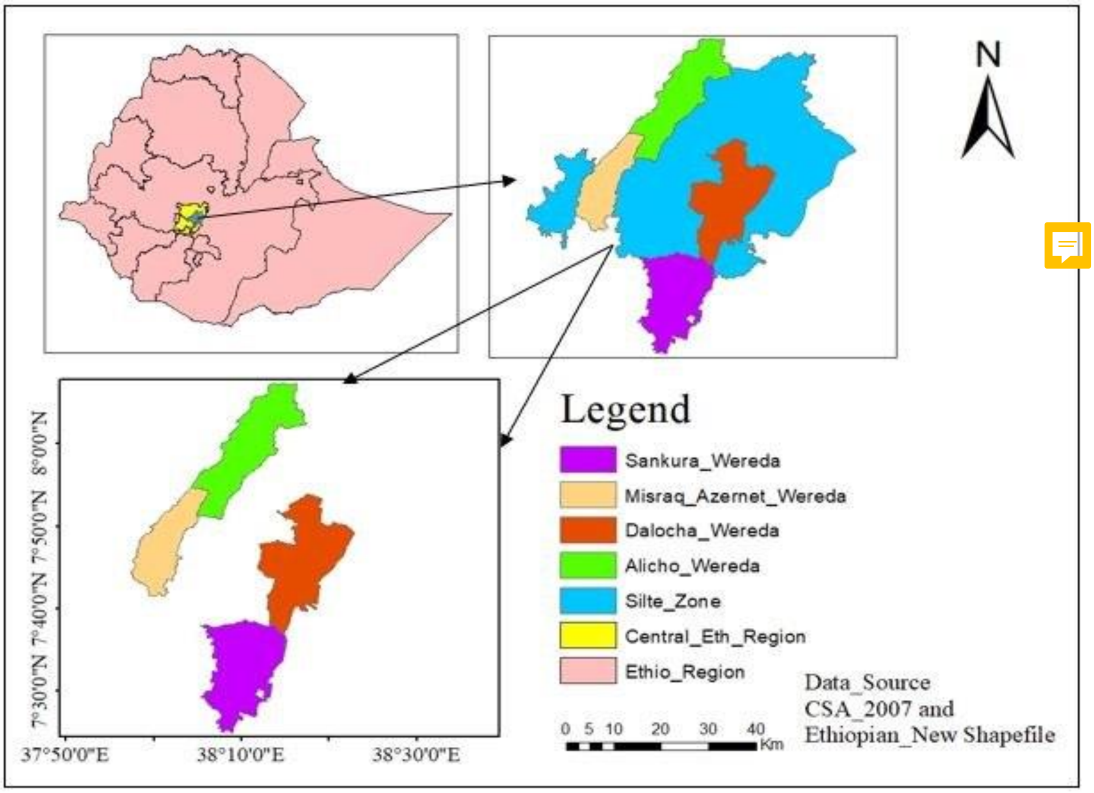

Figure 2: Map of the study area. Source: Arc geography

3.2. Research design

This study employed quantitative research design following (Cresw[illegible]ll, 2009).

3.3. Sampling techniques

Multistage sampling procedures were used; four district from two agro-ecological zones, two kebeles from each sample district. The recruitment period was from 10, May 2024 up to 20, August 2024

3.4. Types, sources, and methods of data collection

A structured questionnaire employed and primary data collected from selected respondents.

3.5. Methods of data analysis

Sustainable livelihood framework (SLF) was adopted as one of the main theoretical underpinnings to lead the analysis of smallholder exposure and adaptation to climate change and its proceeding impact (DFID, 1997). This framework is very helpful to comprehend the link among livelihood, risk (climate change), their adaptation strategies and poverty reduction impact. The SLF consists of five sections; the vulnerability context; livelihood assets; policy,

institutions and procedures; livelihood strategies; and livelihood outcomes. In this study, we refer to vulnerability or exposure as the climate change and its associated shock (erosion and pest infestation). It frequently impacts households in the study area (Bekele et al., 2020; Mathewos et al., 2024).

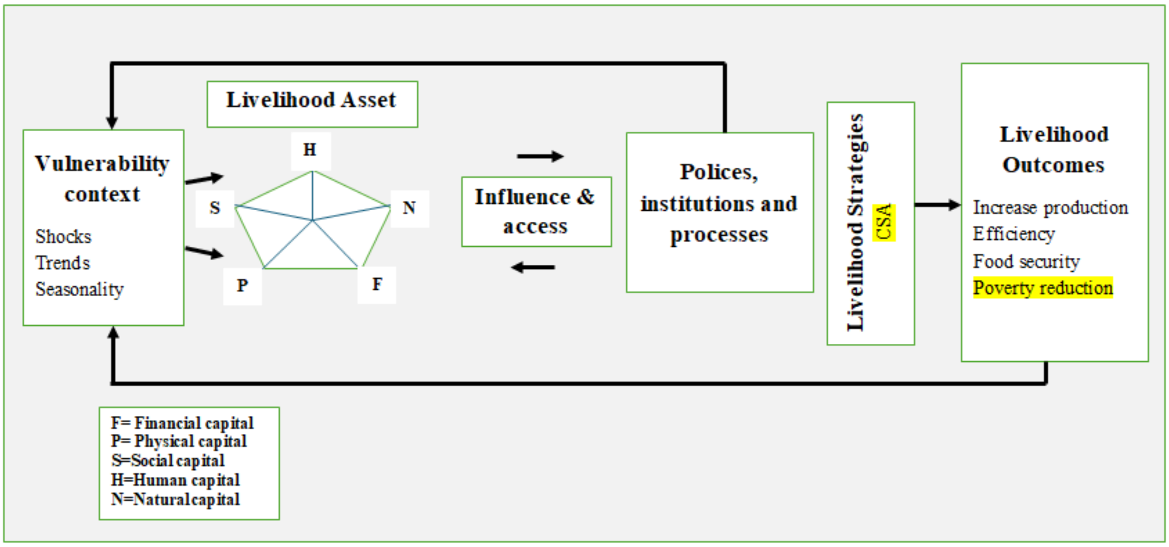

Figure 3: Sustainable livelihood frameworks. Source: Adopted from (Chambers R & Conway, 1992) , with own modification

3.6. Empirical models specification

Generalized structural equation model (GSEM), PSM and 2SLS analytical models are used to analyse the effect of different climate smart agriculture practices adoption on multidimensional poverty. Previous studies adopt the similar method (Abdiwahab et al., 2024; Chan & Wong, 2024, 2025; Macció & Mitchell, 2023; Mohaqeqi Kamal et al., 2024). MPI was used as order categories (non-poor, vulnerable, poor and severely-poor), for GSEM and as continuous variable for PSM and 2SLS.

For the model 2SLS, CSA included as endogenous variable with two instruments (frequency of extension contact and awareness about climate change). Additional exogenous variables are included in the model. Furthermore, endogenous test done to check endogenous problem,

first-stage regression summary statistics that tells us usage of strong instrument and also over identifying restrictions test was done. The 2SLS model formula can be written as:

The First Stage: The endogenous variable, x, is regressed on a set of instrumental variables, z, and other exogenous variables, w, using ordinal least squares (OLS):

$$x_i = \pi_0 + \pi_1 z_{1i} + \cdots \pi_k z_{ki} + \gamma_1 w_{1i} + \cdots + \gamma_m w_{mi} + u_i \text{.....................................} (1)$$

Where x_i is the endogenous variables, z is the instrumental variable, w is the exogenous variable, u is the error term.

The predicted values of x, denoted as $\hat{x}$, are obtained from the first-stage regression:

$$\hat{x}_i = \hat{\pi}_0 + \hat{\pi}_1 z_{1i} + \cdots \hat{\pi}_k z_{ki} + \hat{\gamma}_1 w_{1i} + \cdots + \hat{\gamma}_m w_{mi} \text{...} (2)$$

The second stage: the original equation is re-estimated using OLS, but the endogenous variable, x, is replaced with its predicted values. That is:

$$y_i = \beta_0 + \beta_1 \hat{x}_i + \varepsilon_i \text{ ....} (3)$$

The generalized structural equation model (GSEM) specified as follow:

$$\Pr\left(Y = \frac{y}{z}\right) = \Pr\left(Y^* < k_y - z\right) - \Pr\left(Y^* < k_{y-1} - z\right) \text{..} (4)$$

Where, Y^* is the underlying stochastic component for Y. The distribution for Y^* is determined by the link function. gsem allows logit, probit, and cloglog for the ordinal family. The probit link assigns $Y*$ the standard normal distribution that is synonymous with the probit link for Bernoulli outcomes. So, the latent (unobserved level of poverty) specified as:

$$Y^* = \beta_0 + \beta_1 CSA + \beta'_2 x + \varepsilon \text{...} (5)$$

Where, Y^* are the unobserved categories of multidimensional poverty and ε is error term. The links to observed categories of MPI area as follow:

$$Y = \begin{cases} 1\ if\ Y^* \le z_1 \\ 2\ if\ z_1 < Y^* \le z_2 \\ 3\ if\ z_2 < Y^* \le z_3 \\ . \\ . \\ . \\ k\ if\ z_{k-1} < Y^* \end{cases} \text{...} (6)$$

Where,z_1, z_2, z_3…..z_{k-1} are the cut-off points.

PSM was used to estimate the treatment effect of adopting different CSA practices, Y represented the outcomes (MPI score) dependent on a set of characteristics of farm households denoted as "j" and "i" respectively.

$$Y = \alpha + \tau d_j + \beta x_{ij} + \varepsilon \ldots\ldots\ldots\ldots (7)$$

The average MPI level difference between the treatment (CSA practices) and control (non-users) groups is seen in Equation 8 (τATE). Since unobservable factors may correlate with the use of CSA practices and omitted factors that affect MPI, it is not easy to accurately estimate the impacts. As a result, the Average Effect on the Treated (ATT) is recommended over the Average Treatment Effect (ATE).

$$\tau ATE = E\left[\frac{Y}{X}, d = 1\right] - E[\frac{Y}{X}, d = 0] \ldots\ldots\ldots\ldots (8)$$

Y1 and Y0 in equation 9 represent the MPI scores of CSA practices user against non-users. E [Y0|d = 1] would represent the counterfactual result for the treated groups if they did not use CSA practices.

$$ATE = E[Y_1/d = 1] - E[Y_0/d = 1] \ldots\ldots\ldots\ldots (9)$$

Subsequent matching process was conducted to compare farm households that adopted CSA practices with those that have not, but possess similar characteristics. The propensity score denoted as p(x) and based on a set of characteristics x as outlined by (Rosenbaum & Rubin, 1983), was utilized in this analysis:

$$p(x) = P[\left[P_Y[d = \frac{1}{x}\right] = E[d/x] \ldots\ldots\ldots\ldots (10)$$

Generally there are three assumptions to utilize PSM. The first one is Conditional Independence Assumption (CIA), also referred to as the Un-confoundedness Assumption. This assumption necessitates that the outcome variables (Y0) exhibit independence from the treatment. Second, the presence of common support ensures an appropriate basis for comparison. The third, limit matches to users and non-users who share common support within the distribution of propensity scores (Smith & Todd, 2005).

3.7. Ethical consideration of the study

This study passed through Addis Ababa university institutional review board/IRB for ethical clearance checking the alignment of data collection including inclusion of participant consent and ethical clearance is issued. Furthermore, the data collected with anonymity and confidentiality.

4. RESULTS AND DISCUSSIONS

4.1. Descriptive results

Most of the households in the study area were middle-aged farmers. The average farm size is 1.99 hectare in the study area, which is greater than the average farm size (0.96 hectares) estimated by (Paul & wa Gĩthĩnji, 2018) for high-potential areas, and the national average farm size (0.9 hectares) of smallholders in Ethiopia.

Table 1: Summary of explanatory variables used in the model

Variables	Description	Non-Adopters	Adopter	M[illegible] difference	t-value
Sex	Sex of household head (1 if Male; 0 otherwise)	0	0.77	-0.77	-3.2***
Age	Age of the Household head	55.6	44.1	11.5	2.0**
Marital status	Marital status of HHH	1	1.94	-0.94	-4.3***
Family size	Household size (in adult equivalent)	58.3	72.1	-13.76	-0.88
Farm size	Total landholding size (in hectare)	1	3.58	-2.58	-2.48***
Education	HHH level of schooling	0	3.94	-3.97	-2.02**
Experience	HHH farm experience	30	16.1	14	2.46***
Total income	HH total income (in birr)	6500	17640	-11140	-2.30**
Extension	Frequency of extension contacts	0	3.23	-3.23	-3.04***
Awareness	Awareness of HH about climate change	0	0.63	-0.63	-2.27**
Area of residence	Area of residence of households (1 if Rural, 0 Peri-urban)	0.33	0.49	0.16	-0.55
Agroecology	Agroecology of households where they located (1 if Highland, 0 midland)	0.33	0.56	-0.56	-0.79

Source: Model result, 2024

4.2. Results of econometric models

Hypothesis: Adopting CSA practices decrease MPI level of farm households.

To put the hypothesis to the test and calculate the effect of adopting CSA practices, MPI were used as outcome variable. Different CSA practices adopters were categorized as the treatment group and non-adopters as the control group. Variables that have an equal impact on the control

and treatment groups were chosen to calculate the outcome variables with the assumption that the inclusion of unimportant variables and the exclusion of important variables greatly bias the results (Caliendo & Kopeinig, 2008; Heckman et al., 1997)

Testing the Variance Inflation Factor (VIF) and Contingency Coefficient (C) for the continuous and dummy/categorical variables was done to reduce the impact of multicollinearity. The results showed that the mean values of 1.09 (Table 2) and that the dummy variables had C close to zero (Table 3). Thus, it is determined that the model is free from multicollinearity, giving us the confidence to move forward with our regression.

Table 2: Result of VIF

Variable	VIF	1/VIF
Total farm size	2.65	0.377517
Total income	2.63	0.379946
Labor (ADeq)	1.18	0.845274
Livestock (TLU)	1.18	0.848100
Education	1.02	0.977040
Age	1.02	0.981755
Mean VIF	1.61	

Source: Model result, 2024

Table 3: C test for discrete variables

	Area	Agroecology	Sex	Marital status
Area	1.000			
Agroecology	-0.0201	1.000		
Sex	0.0579	0.0376	1.000	
Marital status	0.0244	-0.0288	-0.0842	1.000

Source: Model result, 2024

4.2.1. Estimation of Generalized structural equation model (GSEM)

The GSEM is a key tool for causal analysis, although it offers tests to assess goodness of fit. The Akaike information criterion (AIC) and Bayesian information criterio[illegible]re used to measure the performance of the model proposed in this study. Table 4 shows the results of the model, AIC and BIC, control variables and different climate smart agriculture practices included in the model.

Table 4: GSEM regression analyses

**MPI (categorical)**	**Coefficient**	**Std. Err.**
**Independent variable**		
Total farm size	0.0876123	0.064131
Education	-0.0051601	0.0224154

Total income	-0.0000572***	0.00002
Area	-0.4690562***	0.1552767
Sex	0.0267309	0.1567544
Marital status	-0.2676608**	0.1233986
Age	-0.1192106*	0.0633536
Age2	0.0013541*	0.0007045
Agroecology	-0.0809196	0.1451989
Labor (ADeq)	-0.0071064**	0.0031601
Livestock (TLU)	0.0050084	0.0247233
**Treatment variable**		
HYV	-0.728471***	0.1864136
SWC	-0.6015962***	0.1914361
Spacing	-0.6503183***	0.1792001
Crop rotation	-0.6554536***	0.1859323
Irrigation	-0.1672021	0.2500056
Crop diversification	-0.7924444***	0.1866686
IPM	-0.5338136***	0.1694423
**MPI group**		
/cut1	-8.439512***	1.496998
/cut2	-8.329911***	1.494774
/cut3	-6.316684***	1.463269
AIC		504.4241
BIC		589.0179

Source: Model result, 2024

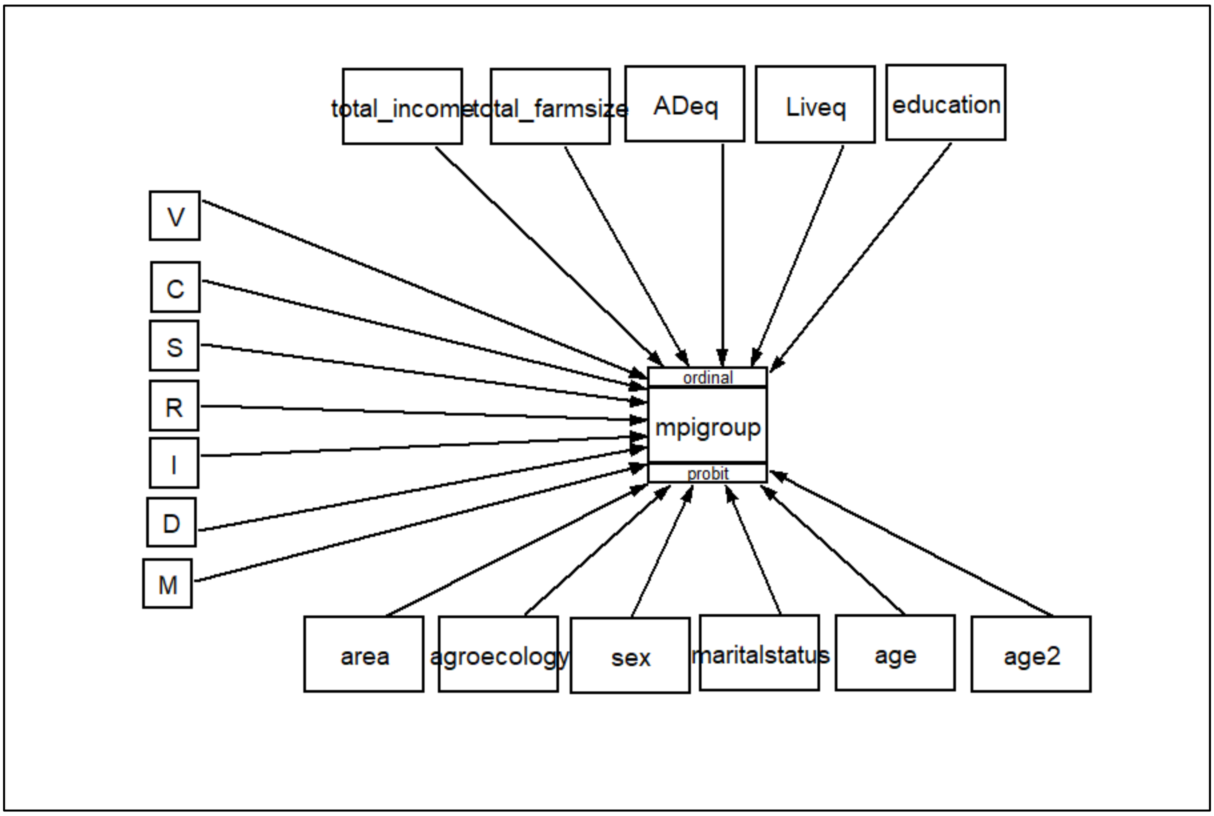

Figure 4: Model GSEM. Source: Model result, 2024

Based on GSEM model, households who adopted improved variety, SWC, spacing, crop rotation, crop diversification and IPM decrease the probability of being in a higher MPI category by 72.8%, 60%, 65%, 65%, 79% and 53% compared to non-adopters respectively. The result is confirmed by the study of (Habtewold, 2021; Islam et al., 2024; Mkupete, 2025; Tariku & Kebede, 2025)

The results of some control variables indicated a significant effect on MPI, supporting previous works that found these variables to be determinants to MPI. For instance, farmers who have more income decreased the probability of being in the higher MPI category by 0.006% than farmers who have less income. Living in a peri-urban area decreases the probability of being in a higher MPI category by 47% compared to rural farmers. Furthermore, marital status, age, and labor decrease the probability of being in higher MPI category by 26.7%, 12% and 0.7% respectively. (Abdiwahab et al., 2024; Chan & Wong, 2024; Mare et al., 2022) found the same result.

For comparison PSM model also used to estimate multidimensional poverty effects of CSA practices adoption. The next section discussed effects of CSA practices adoption on MPI employing PSM.

4.2.2. Estimation of propensity score matching

Ps-test was used to create a balance between the covariates of the two groups. Because of this, the bias percentage that ranged between 0.3 and 89.1 before matching has decreased to 0.1 to 16.4 after matching (Table 6). This shows that the bias percentage has kept well below the 20% critical threshold cut-off point, indicating a minimized imbalance between the treatment and control samples (Rosenbaum & Rubin, 1983). As a result, the covariate balance between the treatment and control samples is greatly enhanced, which can be employed in subsequent estimation processes.

Table 5: Propensity score and covariate matching

**Treatment variable HYV**						
**Variables**	Unmatched/	Mean		% bias	%	**P>/t/**
	Matched	Treated	Controlled		reduction/bias/	
Area	U	0.64706	0.24171	89.1		0.000
	M	0.62147	0.59063	6.8	92.4	0.554

Agroecology	U	0.57843	0.53555	8.6		0.381
	M	0.58192	0.57385	1.6	81.2	0.878
Sex	U	0.67157	0.55924	23.2		0.019
	M	0.64407	0.70354	-12.3	47.1	0.234
Marital	U	1.4706	1.4502	3.4		0.727
status	M	1.4746	1.415	10.1	-192.9	0.342
Education	U	3.6029	3.4265	5.4		0.581
	M	3.5932	3.799	-6.3	-16.6	0.557
Age	U	43.275	44.673	-14.2		0.149
	M	43.446	42.033	14.3	-1.1	0.174
Age2	U	1965.6	2096.2	-14.7		0.136
	M	1982.7	1861.6	13.6	7.2	0.192
Labor (ad.eq)	U	8.238	8.5374	-9.3		0.342
	M	8.1292	8.4671	-10.6	-12.9	0.308
Total farm	U	2.5441	1.3488	71.4		0.000
size	M	2.2486	2.3919	-8.6	88.0	0.94
Total income	U	11311	7708.5	67.1		0.000
	M	10119	10301	-3.4	94.9	0.775
**Treatment variable SWC**						
**Variables**	Unmatched/ Matched	Mean Treated	Controlled	% bias	% reduction/bias/	**P>/t/**
Area	U	0.52482	0.26316	55.4		0.000
	M	0.46025	0.48141	-4.5	91.9	0.644
Agroecology	U	0.57447	0.5188	11.2		0.288
	M	0.57322	0.54601	5.5	51.1	0.550
Sex	U	65248	0.53383	24.3		0.020
	M	0.61506	0.60055	3.0	87.8	0.746
Marital	U	1.4326	1.5188	-14.4		0.167
status	M	1.4644	1.5629	-16.4	-14.3	0.097
Education	U	3.7234	3.0677	20.4		0.055
	M	3.5397	3.6949	-4.8	76.3	0.613
Age	U	43.603	44.797	-12.0		0.251
	M	43.891	45.059	-11.8	2.2	0.197

Age2	U	1996.4	2107.4	-12.3		0.237
	M	2025.9	2125.7	-11.1	10.0	0.221
Labor (ad.eq)	U	8.2832	8.6172	-10.4		0.322
	M	8.3145	7.9082	12.6	-21.6	0.176
Total farm	U	2.2583	1.2538	63.7		0.000
size	M	1.8299	1.8334	-0.2	99.7	0.981
Total income	U	10463	7394.7	63.9		0.000
	M	8788.7	8840.6	-1.1	98.3	0.903
**Treatment variable Spacing**						
**Variables**	Unmatched/		Mean	% bias	%	**P>/t/**
	Matched	Treated	Controlled		reduction/bias/	
Area	U	0.54701	0.30387	50.6		0.000
	M	0.48718	0.51102	-5.0	90.2	0.639
Agroecology	U	0.55983	0.55249	1.5		0.882
	M	0.55385	0.5751	-4.3	-189.4	0.673
Sex	U	0.66239	0.55249	22.6		0.023
	M	0.64615	0.63054	3.2	85.8	0.749
Marital	U	1.4915	1.4199	12.2		0.222
status	M	1.4923	1.4744	3.0	75.0	0.772
Education	U	3.641	3.3481	9.0		0.363
	M	3.5897	3.4327	4.8	46.4	0.626
Age	U	43.568	44.525	-9.7		0.328
	M	43.887	44.571	-6.9	28.5	0.495
Age2	U	1993.7	2081.5	-9.8		0.321
	M	2024.6	2082.8	-6.5	33.7	0.524
Labor (ad.eq)	U	8.1113	8.7508	-20.1		0.043
	M	8.123	8.0064	3.7	81.8	0.722
Total farm	U	2.3509	1.4006	56.6		0.000
size	M	2.0082	1.9765	1.9	96.7	0.858
Total income	U	10767	7814.9	56.3		0.000
	M	9033.3	8927.4	2.0	96.4	0.829
**Treatment variable crop rotation**						
**Variables**	Unmatched/		Mean	% bias	%	**P>/t/**

	Matched	Treated	Controlled		reduction/bias/	
Area	U	0.59375	0.26178	71.1		0.000
	M	0.565	0.56032	1.0	98.6	0.925
Agroecology	U	0.57589	0.53403	8.4		0.393
	M	0.565	0.60839	-8.7	-3.7	0.379
Sex	U	0.66518	0.55497	22.7		0.021
	M	0.65	0.65266	-0.5	97.6	0.956
Marital status	U	1.4911	1.4241	11.3		0.251
	M	1.475	1.4417	5.6	50.3	0.582
Education	U	3.5089	3.5183	-0.3		0.977
	M	3.485	3.2765	6.4	-2118.8	0.513
Age	U	43.589	44.45	-8.7		0.377
	M	43.66	43.097	5.7	34.6	0.579
Age2	U	1999.2	2070.4	-8.0		0.418
	M	2003	1964.7	4.3	46.3	0.674
Labor (ad.eq)	U	8.2665	8.5354	-8.4		0.395
	M	8.264	7.8022	14.4	-71.8	0.166
Total farm size	U	2.4089	1.3822	61.0		0.000
	M	2.2455	2.1414	6.2	89.9	0.590
Total income	U	10848	7874.3	56.0		0.000
	M	9800	9324.3	9.0	84.0	0.388
			**Treatment variable Irrigation**			
**Variables**	Unmatched/	Mean		% bias	%	**P>/t/**
	Matched	Treated	Controlled		reduction/bias/	
Area	U	0.54545	0.43194	22.7		0.209
	M	0.53125	0.52001	2.2	90.1	0.930
Agroecology	U	0.60606	0.55236	10.8		0.552
	M	0.59375	0.59437	-0.1	98.8	0.996
Sex	U	0.69697	0.60733	18.8		0.311
	M	0.6875	0.64475	8.9	52.3	0.722
Marital status	U	1.6061	1.4476	23.7		0.140
	M	1.5938	1.5552	5.8	75.6	0.826
Education	U	4.7273	3.4084	39.9		0.025

	M	4.5625	4.4981	1.9	95.1	0.940
Age	U	39.848	44.343	-50.1		0.012
	M	39.906	40.428	-5.8	88.4	0.801
Age2	U	1647.6	2065.2	-52.5		0.010
	M	1654	1704.1	-6.3	88.0	0.775
Labor (ad.eq)	U	8.7589	8.3584	12.4		0.491
	M	8.6576	8.7245	-2.1	83.3	0.934
Total farm	U	2.3485	1.9008	27.8		0.164
size	M	2.3281	2.1926	8.4	69.7	0.753
Total income	U	10545	9387.4	19.9		0.257
	M	10094	10188	-1.6	91.9	0.950
**Treatment variable crop diversification**						
**Variables**	Unmatched/	Mean		% bias	%	**P>/t/**
	Matched	Treated	Controlled		reduction/bias/	
Area	U	0.64634	0.30677	72.1		0.000
	M	0.61905	0.57817	8.7	88.0	0.476
Agroecology	U	0.56707	0.5498	3.5		0.730
	M	0.57143	0.53119	8.1	-133.0	0.490
Sex	U	0.68902	0.56574	25.6		0.012
	M	0.67347	0.69803	-5.1	80.1	0.652
Marital	U	1.4939	1.4382	9.3		0.350
status	M	1.4762	1.5397	-10.6	-14.0	0.383
Education	U	3.3293	3.6335	-9.4		0.352
	M	3.3129	2.9377	11.6	-23.3	0.306
Age	U	43.665	44.195	-5.3		0.593
	M	43.585	44.244	-6.6	-24.1	0.581
Age2	U	2009.2	2046.9	-4.2		0.674
	M	2004.8	2059.5	-6.1	-45.2	0.610
Labor (ad.eq)	U	8.4773	8.3334	4.5		0.655
	M	8.535	8.1523	12.0	-165.8	0.315
Total farm	U	2.5732	1.5203	60.4		0.000
size	M	2.2517	2.4399	-10.8	82.1	0.391
Total income	U	11561	8119.5	59.8		0.000

	M	10150	10064	1.5	97.5	0.900
**Treatment variable IPM**						
**Variables**	Unmatched/ Matched	Mean Treated	Controlled	% bias	% reduction/bias/	**P>/t/**
Area	U	0.55556	0.31658	49.5		0.000
	M	0.52632	0.51931	1.5	97.1	0.892
Agroecology	U	0.56019	0.55276	1.5		0.880
	M	0.55263	0.58022	-5.5	-271.7	0.589
Sex	U	0.67593	0.54774	26.5		0.007
	M	0.64211	0.6749	-6.8	74.4	0.502
Marital	U	1.4537	1.4673	-2.3		0.815
status	M	1.4474	1.4355	2.0	13.0	0.843
Education	U	3.8472	3.1508	21.6		0.029
	M	3.7842	3.9401	-4.8	77.6	0.653
Age	U	43.519	44.492	-9.9		0.316
	M	43.716	44.038	-3.3	66.9	0.756
Age2	U	1993.9	2073.3	-8.9		0.366
	M	2010.2	2043.7	-3.8	57.8	0.721
Labor (ad.eq)	U	8.3607	8.4223	-1.9		0.845
	M	8.3785	8.4783	-3.1	-62.1	0.759
Total farm	U	2.4282	1.4025	60.9		0.000
size	M	2.0579	2.0459	0.7	98.8	0.945
Total income	U	11023	7804	60.5		0.000
	M	9792.1	9387.7	7.6	87.4	0.471

Source: Model result, 2024

As illustrated in table 7; from kernel matching methods, kernel with bandwidth 0.01 was chosen, by using the values of Ps R^2 and LR chi^2 as indicators for completing the balancing criteria. The assumption that both groups have a similar distribution in covariates after matching is confirmed by the relatively low Ps R^2 0.011, 0.008, 0.003, 0.01, 0.007, 0.0160 and 0.005 for HYV, SWC, spacing, crop rotation, irrigation, crop diversification and IPM respectively. Furthermore, the insignificant LR ch^2 5.26, 5.52, 1.82, 5.48, 0.61, 6.54, and 2.82 after matching for HYV, SWC, spacing, crop rotation, irrigation, crop diversification and

IPM respectively. It is supported by (Habtewold, 2021; Islam et al., 2024; Mkupete, 2025; Tariku & Kebede, 2025)

Table 6: Comparison of the matching estimators

Performance criteria	HYV			Performance criteria	SWC		
	BW (0.05)	**BW (0.01)**	BW (0.1)		BW (0.05)	**BW (0.01)**	BW (0.1)
PsR^2	0.016	**0.011**	0.016	PsR^2	0.037	**0.008**	0.031
LR chi^2	8.850	**5.26**	8.85	LR chi^2	29.27	**5.52**	24.59
$p>chi^2$	0.547	**0.873**	0.547	$p>chi^2$	0.001	**0.854**	0.006
MeanBias	6.0	**8.8**	6.0	MeanBias	14.5	**7.1**	11.5
MedBias	5.9	**9.3**	5.9	MedBias	12.6	**5.1**	12.3
B	29.3*	**24.4**	29.3*	B	46.1*	**21.5**	41.8*
R	2.28*	**1.31**	2.28*	R	1.10	**0.83**	2.10*
%Var	14	**0**	14	%Var	14	**14**	29

Table 6: continued

Performance criteria	Spacing			Performance criteria	Crop rotation		
	BW (0.05)	BW (0.01)	BW (0.1)		BW (0.05)	BW (0.01)	BW (0.1)
PsR^2	0.017	**0.003**	0.012	PsR^2	0.019	**0.01**	0.014
LR chi^2	11.29	**1.82**	7.77	LR chi^2	11.64	**5.48**	8.52
$p>chi^2$	0.335	**0.998**	0.652	$p>chi^2$	0.310	**0.857**	0.578
MeanBias	7.7	**4.1**	6.0	MeanBias	5.6	**6.2**	4.2
MedBias	5.5	**4.0**	5.1	MedBias	5.1	**5.9**	2.3
B	31.1*	**13.7**	25.6*	B	31.8*	**23.4**	27.2*
R	1.37	**1.03**	1.94	R	2.89*	**1.06**	3.76*
%Var	14	**0**	14	%Var	14	**0**	14

Table 6: continued

Performance criteria	Irrigation			Performance criteria	Crop diversification		
	BW (0.05)	BW (0.01)	BW (0.1)		BW (0.05)	BW (0.01)	BW (0.1)

PsR^2	**0.007**	0.008	0.023	PsR^2	0.018	**0.016**	0.014
LR chi^2	**0.61**	0.69	2.08	LR chi^2	8.16	**6.54**	6.24
p>chi^2	**1.00**	1.00	0.996	p>chi^2	0.613	**0.768**	0.795
MeanBias	**4.3**	4.2	9.3	MeanBias	7.4	**8.1**	6.5
MedBias	**4.0**	2.6	10.3	MedBias	6.7	**8.4**	6.8
B	**19.3**	20.5	35.6*	B	31.6*	**29.8***	27.3*
R	**0.87**	0.40*	0.67	R	2.40*	**1.28**	3.00*
%Var	**0**	0	0	%Var	14	**14**	14

Table 6: continued

Performance criteria	IPM		
	BW (0.05)	BW (0.01)	BW (0.1)
PsR^2	0.006	**0.005**	0.007
LR chi^2	3.52	**2.82**	4.38
p>chi^2	0.967	**0.985**	0.929
MeanBias	3.9	**3.9**	4.6
MedBias	3.5	**3.5**	3.8
B	18.0	**17.1**	20.0
R	2.00*	**1.73**	2.54*
%Var	0	**14**	14

Source: Model result, 2024

According to the Minima and Maxima criterion, observations with a propensity score smaller than and larger than the opposing group were eliminated while determining the common support region (Caliendo & Kopeinig, 2008) As a result, the range of common support is between (0.138 and 0.970), (0.325 and 0.995), (0.261 and 0.949), (0.207 and 0.956), (0.092 and 0.913), (0.0008 and 0.456) and (0.267 and 0.953) for HYV, SWC, spacing, crop rotation, crop diversification, irrigation and IPM respectively and any households outside of this range were excluded from the matching process. The result is confirmed by (Habtewold, 2021; Islam et al., 2024; Mkupete, 2025; Tariku & Kebede, 2025)

Table 7: Distribution of estimated propensity scores

Variable	Group	Obs.	Mean	SD	Min	Max

Pscore (HYV)	Total	415	0.4923984	0.2428627	0.1389612	0.9700462
	Treated	204	0.6127755	0.238342	0.1389612	0.9700462
	Controlled	211	0.3760147	0.1835203	0.1471128	0.9646568
Pscore (SWC)	Total	415	0.6798698	0.1686818	0.3198355	0.9957257
	Treated	282	0.7210681	0.1696418	0.3259327	0.9957257
	Controlled	133	0.5925169	0.1290865	0.3198355	0.9539493
Pscore (Spacing)	Total	415	0.5636643	0.1744424	0.2562972	0.9493206
	Treated	282	0.6028278	0.1788728	0.2611426	0.9493206
	Controlled	133	0.4806257	0.1307828	0.2562972	0.9473756
Pscore (Crop rotation)	Total	415	0.5386952	0.2021016	0.207716	0.9566356
	Treated	282	0.5825777	0.2050881	0.207716	0.9566356
	Controlled	133	0.445651	0.1604026	0.2169875	0.9387753
Pscore (Crop diversification)	Total	415	0.3942165	0.2086554	0.0927305	0.9136933
	Treated	282	0.4359596	0.2182523	0.0927305	0.9136933
	Controlled	133	0.3057086	0.1533941	0.0960882	0.9046335
Pscore (Irrigation)	Total	415	0.0795706	0.0681301	0.0008883	0.4560932
	Treated	282	0.0870917	0.0734612	0.0008883	0.4560932
	Controlled	133	0.0636237	0.0518931	0.0017508	0.2767974
Pscore (IPM)	Total	415	0.5193335	0.1801154	0.2607841	0.9531466
	Treated	282	0.5612729	0.1893624	0.2679787	0.9531466
	Controlled	133	0.4304094	0.1170007	0.2607841	0.9034773

Source: Model result, 2024

Figure 5 depicts the propensity score of the distribution density of the treatment and control groups graphically. This indicates that the balance has been successfully attained because the p-score is fairly distributed between the treatment and control groups.

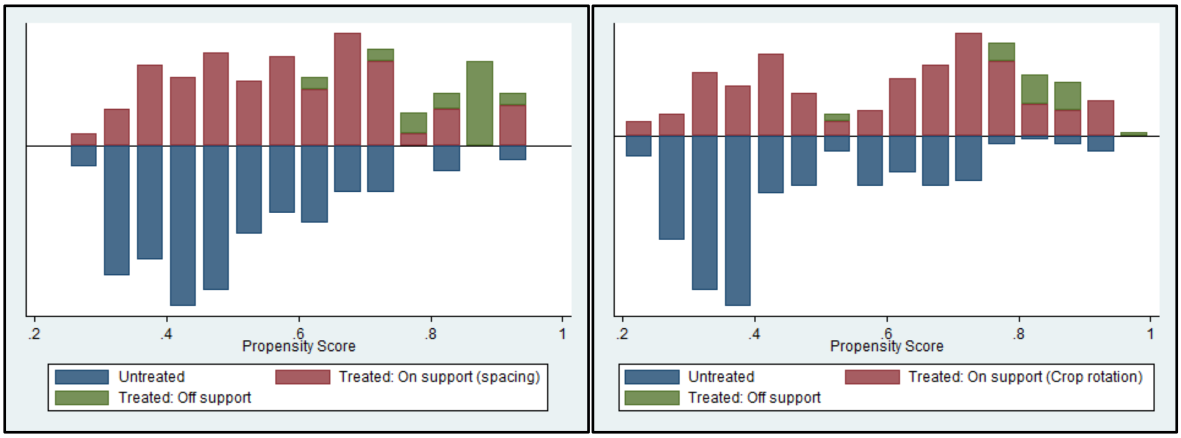

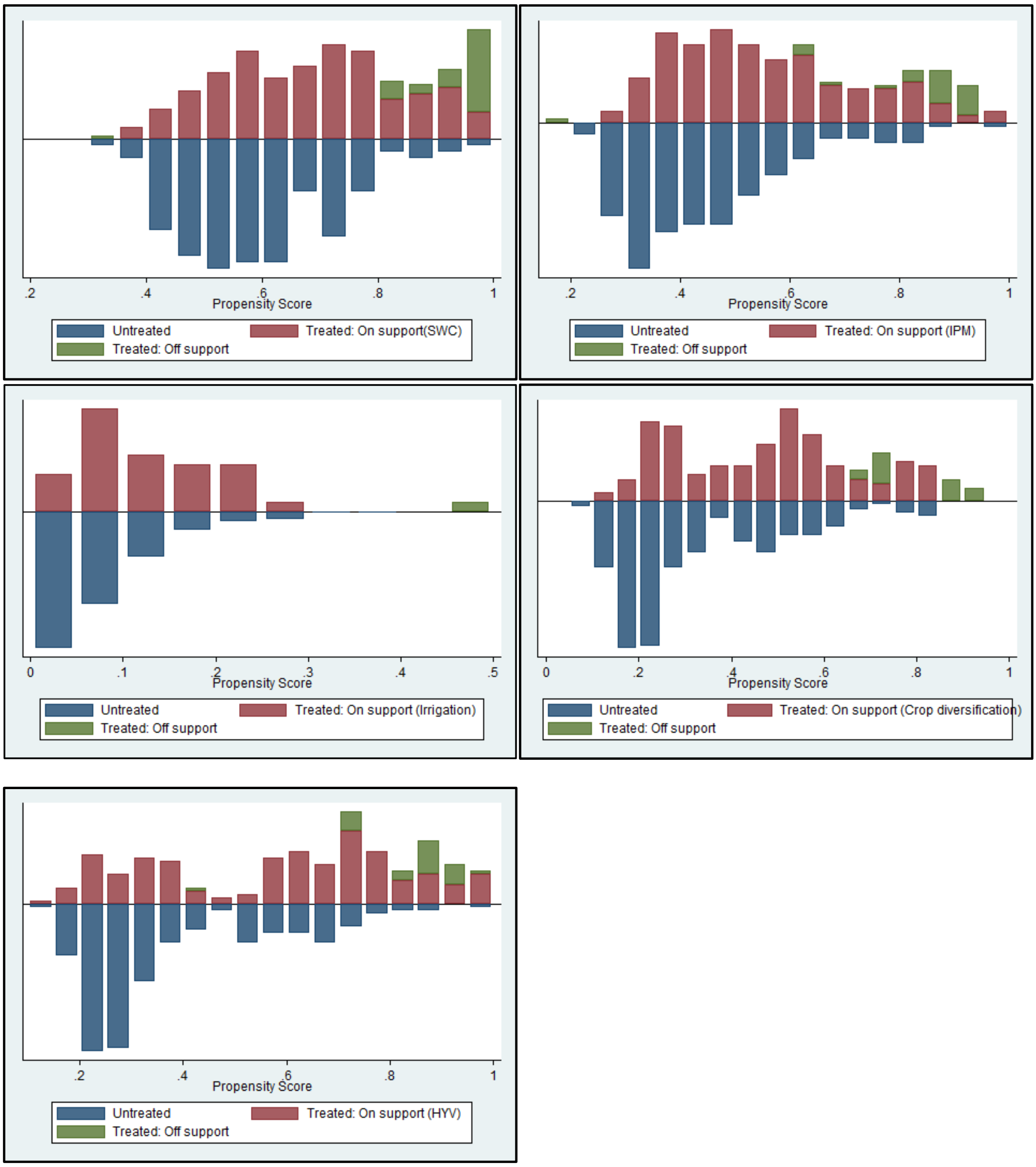

Figure 5: Distribution of propensity score generated from Kernel Matching Source: Model result, 2024

4.2.3. Estimation of treatment effect on the treated

As Tables 8 show the results of the impacts of the use of HYV, SWC, Spacing, crop rotation, Irrigation, crop diversification and IPM on the outcome variables of multidimensional poverty. According to the Kernel estimate, farm who adopted CSA practices significantly minimize MPI level by 0.20, 0.13, 0.12, 0.17, 0.04, 0.04 and 0.17 for HYV, SWC, spacing, crop rotation, irrigation, crop diversification and IPM adopters compared with non-adopters respectively. This finding supported by the findings of (Habtewold, 2021; Islam et al., 2024; Mkupete, 2025; Tariku & Kebede, 2025)

Table 8: CSA practices adoption result of average treatment effect on the treated (ATT)

CSA practices	DV	Sample	Treated	Controlled	Difference	S.E	T-stat
HYV	MPI	Unmatched	0.585457514	0.836334906	-0.250877392	0.018130887	-13.84
	score	ATT	0.585457514	0.785620911	-0.200163397	0.022495792	-8.90
SWC	MPI	Unmatched	0.655555553	0.834837085	-0.179281532	0.021779364	-8.23
	score	ATT	0.655555553	0.783569734	-0.128014181	0.025934461	-4.94
Spacing	MPI	Unmatched	0.638319086	0.80957642	-0.171257333	0.020443784	-8.38
	score	ATT	0.638319086	0.760968656	-0.12264957	0.023294732	-5.27
Crop	MPI	Unmatched	0.608779759	0.835253048	-0.226473288	0.018969962	-11.94
rotation	score	ATT	0.608779759	0.777380948	-0.168601188	0.023756353	-7.10
Irrigation	MPI	Unmatched	0.61313131	0.721640484	-0.108509174	0.040178637	-2.70
	score	ATT	0.61313131	0.657575756	-0.044444446	0.06483768	-0.69
Crop	MPI	Unmatched	0.5550813	0.816201853	-0.261120553	0.018384886	-14.20
diversification	score	ATT	0.568253967	0.773330955	-0.205076988	0.023351161	-8.78
IPM	MPI	Unmatched	0.615895059	0.818425454	-0.202530395	0.019557042	-10.36
	score	ATT	0.615895059	0.790586414	-0.174691354	0.022618323	-7.72

Source: Model result, 2024

4.2.4. Sensitivity analysis

Sensitivity analysis is conducted to evaluate how reliable or consistent the PSM results are (Caliendo & Kopeinig, 2008; Liu et al., 2013). Based on this, the robustness of hidden bias among the two outcome variables of the study is shown in Table 9. The lowest critical value (Γ) that includes zero for the productivity outcome variable is 8.8 (95% confidence interval). This result suggested that the two groups should have differed by up to 780% (e^{Υ} =8.8) in terms of unobserved variables if it was to be sensitive to unobserved factors, which is unlikely under normal circumstances. This result has also indicated that for the productivity and income estimated at various levels of critical values e^{Υ}, the p-critical values are significant, informing that important covariates that affected the outcomes have been taken into account. Overall, the hidden bias magnitude confirms the hypothesis that the findings of significant differences in MPI level between users and non-users are insensitive to hidden biases; as a result, the positive impacts found are attributed to the use of different CSA practices.

Table 9: Rosenbaum bounds sensitivity analysis

*Gamma (hidden bias)	Significance level		Hodges-Lehmann point estimate		Confidence interval (95%)	
	upper bound (sig+)	lower bound sig-	upper bound (t-hat+)	lower bound (t-hat-)	upper bound (CI+)	lower bound (CI-)
Outcome variables MPI effect of HYV						
1	0	0	-0.15	-0.15	-0.216667	-0.116667
2	0	1.6e-11	-0.283333	-0.083333	-0.316667	-0.066667
**6.6**	**0**	**0.025777**	**-0.4**	**-0.016666**	**-0.45**	**3.8e-07**
Outcome variables MPI effect of SWC						
1	0	0	-0.1	-0.1	-0.116667	-0.066667
2	0	0.000032	-0.166667	-0.05	-0.216667	-0.016667
**2.9**	**0**	**0.036286**	**-0.233333**	**-0.016667**	**-0.266667**	**4.9e-07**
Outcome variables MPI effect of Spacing						
1.5	0	0.000401	-0.133334	-0.033333	-0.2	-0.016666
1.6	0	0.001514	-0.15	-0.033333	-0.216667	-0.016666
**2**	**0**	**0.049856**	**-0.2**	**-0.016666**	**-0.25**	**4.7e-07**
Outcome variables MPI effect of crop rotation						
1	0	0	-0.133334	-0.133334	-0.166667	-0.1
2	0	5.0e-09	-0.233334	-0.083333	-0.283333	-0.05
**4.5**	**0**	**0.025583**	**-0.333334**	**-0.033333**	**-0.383334**	**3.2e-07**
Outcome variables MPI effect of Irrigation						
1.9	0.037561	0.930385	-0.116666	0.066666	-0.35	0.233333
2	0.027673	0.946584	-0.116667	0.083333	-0.35	0.233333
**4.4**	**0.000012**	**0.999943**	**-0.316667**	**0.2**	**-0.616666**	**0.4**
Outcome variables MPI effect Crop diversification						
1	0	0	-0.183334	-0.183334	-0.233333	-0.133333
2	0	7.4e-09	-0.283333	-0.1	-0.333334	-0.083333
**5.5**	**0**	**0.027614**	**-0.4**	**-0.033333**	**-0.45**	**3.9e-07**
Outcome variables MPI effect of IPM						
1	1.1e-12	1.1e-12	-0.116667	-0.116667	-0.15	-0.083333
2	0	0.000558	-0.216666	-0.05	-0.283334	-0.016667
**2.7**	**0**	**0.037504**	**-0.266667**	**-0.016667**	**-0.316667**	**2.8e-07**

4.2.5. Estimation of Two stage least square (2SLS)

As Tables 10 show the results of the effect of the use of HYV, SWC, Spacing, crop rotation, Irrigation, crop diversification and IPM on the outcome variables of multidimensional poverty.

Farm who adopted CSA practices significantly minimize MPI level by 0.15, 0.25, 0.25, 0.15, 0.18, 0.12 and 0.45 for HYV, SWC, spacing, crop rotation, irrigation, crop diversification and IPM adopters compared with non-adopters respectively. This finding supported by the findings of (Habtewold, 2021; Islam et al., 2024; Mkupete, 2025; Tariku & Kebede, 2025)

Table 10: Two stage least square (2SLS) model output

**MPI score (outcome variable)**	**Coefficient**	**Std. Err.**	**P>z**
**HYV (treatment variable)**	-.1471345***	.0450631	0.001
Area	-.1484687***	.0217406	0.000
Agroecology	-.0141885	.015187	0.350
Sex	.0046432	.0158653	0.770
Marital status	-.0277207**	.0133337	0.038
Education	.0011372	.002306	0.622
Age	-.0051056	.0063504	0.421
Age2	.0000632	.0000702	0.368
TLU	.0020789	.0025555	0.416
Labor	-.0007673**	.0003165	0.015
Total farm size	.0269427***	.0072225	0.000
Total income	-.0000195***	2.19e-06	0.000
cons	1.158031***	.1423797	0.000
**MPI score (outcome variable)**	**Coefficient**	**Std. Err.**	**P>z**
**SWC (treatment variable)**	-.2528677***	.0814086	0.002
Area	-.1545883***	.0227003	0.000
Agroecology	-.0074065	.0178536	0.678
Sex	.0088454	.0183749	0.630
Marital status	-.04387***	.0159185	0.006
Education	.0036243	.0027335	0.185
Age	-.0038078	.0073098	0.602
Age2	.0000502	.0000809	0.535
TLU	.001712	.0029509	0.562
Labor	-.0009264**	.000364	0.011
Total farm size	.0291794***	.0084595	0.001
Total income	-.0000191***	2.53e-06	0.000
cons	1.243923***	.1676482	0.000
**MPI score (outcome variable)**	**Coefficient**	**Std. Err.**	**P>z**
**spacing (treatment variable)**	-.2544787***	.0779567	0.001
Area	-.1536873***	.0227646	0.000
Agroecology	-.0180189	.0176622	0.308
Sex	.0123399	.0189262	0.514
Marital status	-.0160766	.0162178	0.322
Education	.0018242	.0027062	0.500
Age	-.0061706	.0074632	0.408
Age2	.0000713	.0000824	0.387
TLU	-.0000468	.0031594	0.988
Labor	-.0009553***	.0003726	0.010
Total farm size	.0275629***	.0084384	0.001

Total income	-.000018***	2.66e-06	0.000
cons	1.256071***	.1715335	0.000
**MPI score (outcome variable)**	**Coefficient**	**Std. Err.**	**P>z**
**Crop rotation (treatment variable)**	-.152436***	.040342	0.000
Area	-.1562303***	.0192205	0.000
Agroecology	-.0149492	.0152714	0.328
Sex	.0076149	.0160895	0.636
Marital status	-.0224391*	.0136051	0.099
Education	.0005855	.0023397	0.802
Age	-.0084854	.0064772	0.190
Age2	.000102	.0000715	0.154
TLU	.0023017	.0025594	0.368
Labor	-.0007562**	.0003197	0.018
Total farm size	.0270435***	.0072283	0.000
Total income	-.00002***	2.18e-06	0.000
cons	1.23577***	.1464581	0.000
**MPI score (outcome variable)**	**Coefficient**	**Std. Err.**	**P>z**
**Irrigation (treatment variable)**	-.996119*	.5316671	0.061
Area	-.1785858***	.0323248	0.000
Agroecology	.0072097	.0333347	0.829
Sex	.0151193	.0321114	0.638
Marital status	.0307086	.0417534	0.462
Education	.0110768	.0068314	0.105
Age	-.0017896	.0126264	0.887
Age2	-.0000175	.0001456	0.904
TLU	.0072344	.0052814	0.171
Labor	-.000801	.0006219	0.198
Total farm size	.0209403	.0136677	0.125
Total income	-.0000196***	4.28e-06	0.000
cons	1.029698***	.2841021	0.000
**MPI score (outcome variable)**	**Coefficient**	**Std. Err.**	**P>z**
**Crop diversification (treatment variable)**	-.1222782***	.0354937	0.001
Area	-.165749***	.0178794	0.000
Agroecology	-.0204649	.0146978	0.164
Sex	.008035	.0156365	0.607
Marital status	-.0249901*	.013117	0.057
Education	-.0000341	.0022982	0.988
Age	-.0087402	.0063037	0.166
Age2	.0001071	.0000697	0.124
TLU	.0038605	.0024538	0.116
Labor	-.0008197***	.0003091	0.008
Total farm size	.0235269***	.0068537	0.001
Total income	-.0000193***	2.15e-06	0.000
cons	1.207196***	.1410483	0.000

MPI score (outcome variable)	Coefficient	Std. Err.	P>z
**IPM (treatment variable)**	-.4569242***	.1675807	0.006
Area	-.121709***	.0358308	0.001
Agroecology	-.014498	.0217335	0.505
Sex	.0268604	.0247217	0.277
Marital status	-.0298824	.0191303	0.118
Education	.0064673*	.0037777	0.087
Age	-.0149542	.0098121	0.127
Age2	.0001688	.0001075	0.116
TLU	.004355	.0036098	0.228
Labor	-.0013937***	.0004977	0.005
Total farm size	.0353581***	.0113786	0.002
Total income	-.0000157***	3.61e-06	0.000
cons	1.474593***	.2410756	0.000

5. Conclusion and recommendations

5.1. Conclusion

This study investigated the pathways through which Climate-Smart Agriculture (CSA) adoption affects multidimensional poverty among rural and peri-urban households, using Generalized Structural Equation Model (GSEM), PSM and 2SLS. By treating both CSA practices separately as dummy variables and multidimensional poverty as continuous outcome variable.

The result from the PSM showed that use the all CSA practices drop down the level of MPI from 0.04-0.20. The findings reveal that CSA adoptions are significantly associated with lower levels of multidimensional poverty. Similarly, the results from GSEM that uses MPI as categorical outcome variable found low probability of CSA practices adopters to be in higher MPI category compared with non-adopters. Similarly the finding from 2SLS shows multidimensional poverty effects of different CSA practices adoption. Therefore, the government should focus on dissemination of different CSA practices to struggle the challenges of climate change, increase production and productivity so as to minimize multidimensional poverty. Extension services have a role to do so. Future research could build on this study's findings to determine whether adopting the different CSA practices has varied consequences on different agro-ecology and location.

5.2. Recommendations

- ✓ Strengthen Resilience: CSA initiatives should explicitly aim to build household resilience to climate change and achieving sustainable development and poverty reduction.
- ✓ Promote Higher-Intensity CSA Adoption: Since poverty reduction outcomes are strongest among households who adopted CSA prctices, programs should move beyond pilot activities to ensure broad, sustained adoption across communities, with support for training, inputs, and credit.
- ✓ Future studies should focus on accounting for endogeneity and feedback loops between technology adoption and poverty outcomes to capture the effect of poverty on CSA adoption.

References

Abdiwahab, B., Menza, M., & Mohamed, A. (2024). Multidimensional Poverty and its Determinants in Somalia: A Household Level Analysis. *International Journal of Developing Country Studies*, *6*(1). https://doi.org/10.47941/ijdcs.2108

Adimassu, Z., Tibebe, D., Tamene, L., & Abera, W. (2025). Evaluating the effects of Climate Smart Agricultural (CSA) practices on productivity, adaptation, and mitigation indicators in Ethiopia: A meta-analysis approach. *Heliyon*, *11*(4). https://doi.org/10.1016/j.heliyon.2025.e42796

Ali, H., Menza, M., Hagos, F., Haileslassie, A., Ali, H., Menza, M., Hagos, F., & Haileslassie, A. (2023). Impact of climate-smart agriculture adoption on food security and multidimensional poverty of rural farm households in the Central Rift Valley of Ethiopia. *Agriculture & Food Security 2022 11:1*, *11*(1). https://doi.org/10.1186/s40066-022-00401-5

Bekele, B., Gemi, Y., Bekele, B., & Gemi, Y. (2020). Soil erosion risk and sediment yield assessment with universal soil loss equation and GIS: in Dijo watershed, Rift valley Basin of Ethiopia. *Modeling Earth Systems and Environment 2020 7:1*, *7*(1). https://doi.org/10.1007/s40808-020-01017-z

Belay, A., Mirzabaev, A., Recha, J. W., Oludhe, C., Osano, P. M., Berhane, Z., Olaka, L. A., Tegegne, Y. T., Demissie, T., Mutsami, C., & Solomon, D. (2023). Does climate-smart agriculture improve household income and food security? Evidence from Southern Ethiopia. *Environment, Development and Sustainability*. https://doi.org/10.1007/s10668-023-03307-9

Bollen, K. A. (1996). An alternative two stage least squares (2SLS) estimator for latent variable equations. *Psychometrika 1996 61:1*, *61*(1). https://doi.org/10.1007/BF02296961

Caliendo, M., & Kopeinig, S. (2008). SOME PRACTICAL GUIDANCE FOR THE IMPLEMENTATION OF PROPENSITY SCORE MATCHING. *Journal of Economic Surveys*, *22*(1). https://doi.org/10.1111/j.1467-6419.2007.00527.x

Chan, S. M., & Wong, H. (2024). Measurement and determinants of multidimensional poverty: the case of Hong Kong. *Journal of Asian Public Policy*, 1-21. https://doi.org/10.1080/17516234.2024.2325857 (Journal of Asian Public Policy)

Chan, S. M., & Wong, H. (2025). Measurement and determinants of multidimensional poverty: the case of Hong Kong. *Journal of Asian Public Policy*, 1-21. https://doi.org/10.1080/17516234.2024.2325857

Creswell, J. W. (2009). *Research design: Qualitative, quantitative, and mixed methods approaches*. SAGE. https://www.ucg.ac.me/skladiste/blog_609332/objava_105202/fajlovi/Creswell.pdf

DFID. (1997). *Sustainable livelihoods guidance sheets*. UK Retrieved from https://www.livelihoodscentre.org/documents/114097690/114438878/Sustainable%2Blivelihoods%2Bguidance%2Bsheets.pdf/594e5ea6-99a9-2a4e-f288-cbb4ae4bea8b?t=1569512091877&utm_source=chatgpt.com

Habtewold, T. M. (2021). Impact of climate-smart agricultural technology on multidimensional poverty in rural Ethiopia. *Journal of Integrative Agriculture*, *20*(4). https://doi.org/10.1016/S2095-3119(21)63637-7

Heckman, J. J., Ichimura, H., & Todd, P. E. (1997). Matching As An Econometric Evaluation Estimator: Evidence from Evaluating a Job Training Programme. *The Review of Economic Studies*, *64*(4). https://doi.org/10.2307/2971733

Islam, M. K., Farjana, F., Islam, M. K., & Farjana, F. (2024). Impact of climate-smart agriculture practices on multidimensional poverty among coastal farmers in Bangladesh. *Communications Earth & Environment 2024 5:1*, *5*(1). https://doi.org/10.1038/s43247-024-01570-w

Liu, J., Kwan, H. K., Lee, C., & Hui, C. (2013). Work-to-Family Spillover Effects of Workplace Ostracism: The Role of Work-Home Segmentation Preferences. *Human Resource Management*, *52*(1). https://doi.org/10.1002/hrm.21513

Macció, J., & Mitchell, A. (2023). Multidimensional Poverty Measurement in Segregated Cities: Evidence from the City of Buenos Aires. *Revista Desarrollo y Sociedad*(93). https://doi.org/10.13043/DYS.93.3

Mare, Y., Gecho, Y., & Mada, M. (2022). Determinants of multidimensional rural poverty in Burji and Konso area, Southern Ethiopia. *Cogent Social Sciences*, *8*(1). https://doi.org/10.1080/23311886.2022.2068757

Mathewos, M., Wosoro, D., & Wondrade, N. (2024). Quantification of soil erosion and sediment yield using the RUSLE model in Boyo watershed, central Rift Valley Basin of Ethiopia. *Heliyon*, *10*(10). https://doi.org/10.1016/j.heliyon.2024.e31246

Midjangninou, S. L. A., Bonou, A., Kpenavoun Chogou, S., Midjangninou, S. L. A., Bonou, A., & Kpenavoun Chogou, S. (2024). Impact of climate-smart agriculture on the income and food security of cassava-producing households in the Savalou commune in Benin. *Discover Sustainability 2024 5:1*, *5*(1). https://doi.org/10.1007/s43621-024-00451-8

Mkupete, M. J. (2025). Is agricultural technology adoption effective in reducing multidimensional poverty? Panel data evidence from Tanzania. *Food Policy*, *135*. https://doi.org/10.1016/j.foodpol.2025.102904

Mohaqeqi Kamal, S. H., Basakha, M., & Alkire, S. (2024). Multidimensional poverty index: a multilevel analysis of deprivation among Iranian older adults. *Ageing and Society*, *44*(2), 337-356. https://doi.org/10.1017/S0144686X2200023X (Ageing and Society)

Muthén, B. (1984). A general structural equation model with dichotomous, ordered categorical, and continuous latent variable indicators. *Psychometrika 1984 49:1*, *49*(1). https://doi.org/10.1007/BF02294210

Nkumulwa, H. O., & Pauline, N. M. (2021). Frontiers | Role of Climate-Smart Agriculture in Enhancing Farmers' Livelihoods and Sustainable Forest Management: A Case of Villages Around Songe-Bokwa Forest, Kilindi District, Tanzania. *Frontiers in Sustainable Food Systems*, *5*. https://doi.org/10.3389/fsufs.2021.671419

Paul, M., & wa Gĩthĩnji, M. (2018). Small farms, smaller plots: land size, fragmentation, and productivity in Ethiopia. *The Journal of Peasant Studies*, *45*(4), 757-775. https://doi.org/10.1080/03066150.2016.1278365

Radeny, M., Rao, E. J. O., Ogada, M. J., Recha, J. W., Solomon, D., Radeny, M., Rao, E. J. O., Ogada, M. J., Recha, J. W., & Solomon, D. (2022). Impacts of climate-smart crop varieties and livestock breeds on the food security of smallholder farmers in Kenya. *Food Security 2022 14:6*, *14*(6). https://doi.org/10.1007/s12571-022-01307-7

Rogers, E. M. (1962). *Diffusion of Innovations. Free Press, New York. - References - Scientific Research Publishing*. https://www.scirp.org/reference/referencespapers?referenceid=2008938

Rosenbaum, P. R., & Rubin, D. B. (1983). The central role of the propensity score in observational studies for causal effects. *Biometrika*, *70*(1). https://doi.org/10.1093/biomet/70.1.41

Samuel, J., Rama Rao, C. A., Pushpanjali, Anshida Beevi, C. N., Raju, B. M. K., Amarender Reddy, A., Nagarjuna Kumar, R., Reddy, A. G. K., Singh, V. K., Prabhakar, M., Siva, G. S., & Teggelli, R. G. (2024). Frontiers | Enhancing farm income resilience through climate smart agriculture in drought-prone regions of India. *Frontiers in Water*, *6*. https://doi.org/10.3389/frwa.2024.1327651

Sen, A. (1976). Poverty: An Ordinal Approach to Measurement. *Econometrica*, *44*(2), 219-219. https://doi.org/10.2307/1912718

Smith, J. A., & Todd, P. E. (2005). Does matching overcome LaLonde's critique of nonexperimental estimators? *Journal of Econometrics*, *125*(1-2). https://doi.org/10.1016/j.jeconom.2004.04.011

Tariku, G. D., & Kebede, S. A. (2025). Climate-smart agricultural practices and its implication in Ethiopia: a systematic review. *International Journal of Climate Change Strategies and Management*, *17*(1). https://doi.org/10.1108/IJCCSM-01-2024-0012

Teklu, A., Simane, B., & Bezabih, M. (2022). Effectiveness of Climate-Smart Agriculture Innovations in Smallholder Agriculture System in Ethiopia. *Sustainability (Switzerland)*, *14*(23). https://doi.org/10.3390/su142316143

Teklu, A., Simane, B., Bezabih, M., Teklu, A., Simane, B., & Bezabih, M. (2022). Effectiveness of Climate-Smart Agriculture Innovations in Smallholder Agriculture System in Ethiopia. *Sustainability 2022, Vol. 14, Page 16143*, *14*(23). https://doi.org/10.3390/su142316143

Tesfaye, W., Blalock, G., & Tirivayi, N. (2021). Climate-Smart Innovations and Rural Poverty in Ethiopia: Exploring Impacts and Pathways. *American Journal of Agricultural Economics*, *103*(3). https://doi.org/10.1111/ajae.12161

Zerssa, G., Feyssa, D., Kim, D.-G., Eichler-Löbermann, B., Zerssa, G., Feyssa, D., Kim, D.-G., & Eichler-Löbermann, B. (2021). Challenges of Smallholder Farming in Ethiopia and Opportunities by Adopting Climate-Smart Agriculture. *Agriculture 2021, Vol. 11, Page 192*, *11*(3). https://doi.org/10.3390/agriculture11030192

Ethical clearance

Click here to access/download
Supporting Information
Ethical certificate.pdf
