# Supplementary material for: Multidimensional poverty reduction effect of climate smart agriculture practices among rural households in Siltie Zone, Central Ethiopia
Source: PLoS One. 2026 May 4;21(5):e0348583. doi: 10.1371/journal.pone.0348583 (PMC13138676; doi:10.1371/journal.pone.0348583)
Supplement: Attachment — Submitted filename: REVIEWERs comments PONE.docx [file pone.0348583.s001.docx]

**REVIEWER’S COMMENTS FOR AUTHORS**

The manuscript addresses an important and policy-relevant topic by examining the relationship between climate-smart agriculture (CSA) practices and multidimensional poverty among rural households. The study has the potential to contribute to the growing literature on sustainable CS technologies and rural welfare outcomes. However, several aspects of the manuscript require clarification and strengthening:

**Methodology and Empirical models specification**

1. What is Kebele? Authors should give a short definition of it for clarity. What do you mean by “risk (climate change)”? In the SLF, why is the CSA and poverty reduction coloured in yellow?
2. In 3.6, authors should properly justify why GSEM, PSM, and 2SLS were appropriate for this study. It is not enough that previous studies used it. Why is it appropriate for your own study? What distinct analytical role does each model play? For example, is GSEM used to model the ordered structure of poverty categories, while PSM and 2SLS are used to estimate causal effects? If so, this rationale should be explicitly articulated.
3. A clear statement should be provided. What are the additional exogenous variables? Where are the exogenous variables? You mentioned two endogenous variables, but only one was expressed as equations. Please mention all these variables clearly.
4. Where are the CSA practices? Please mention them before they appear in the results section.
5. Can the Authors put their ethical consideration at the end of the work?
6. Are the three models applied as robustness checks, or are they intended to address different econometric concerns, such as selection bias and endogeneity?
7. The dependent variable, Multidimensional Poverty Index (MPI), is treated inconsistently across the models. The authors indicate that MPI is used as ordered categories (non-poor, vulnerable, poor, and severely poor) in the GSEM framework and as a continuous variable in the PSM and 2SLS models. How exactly was the MPI constructed, and what thresholds were used to categorize households into the four poverty groups? The methodology section should provide a clear explanation of the MPI construction process, indicator weighting, and poverty cut-off values, as these are critical for interpreting the results.
8. The validity of the instrumental variables used in the 2SLS model requires stronger justification. The authors propose the frequency of extension contact and climate change awareness as instruments for CSA adoption. While these variables may plausibly influence adoption decisions, it is not immediately clear that they satisfy the exogeneity condition, which requires that the instruments affect multidimensional poverty only through CSA adoption. For instance, extension contact may directly influence household productivity, income, or access to information, which could independently affect poverty outcomes. The authors should therefore provide a stronger theoretical justification for that and explain why these variables are valid instruments in this context.
9. The authors mention the inclusion of additional exogenous variables in the model but do not explicitly identify them. All these variables should be clearly stated in the
10. Authors should explicitly present the first-stage equation showing how CSA adoption is regressed on the instrumental variables and other exogenous controls.
11. The authors should strengthen their explanation of why ATT is the preferred estimator in this study with supporting literature.

**Results and Discussions**

1. For your descriptive results, what are the implications that most households were of middle age? A short statement explaining the meaning should be written.
2. Round up or round down your figures for age and family size. For instance, for age, 55.6 is approximately 56, and for family size, 58.3 is approximately 58
3. The Authors said that “Different CSA practices adopters were categorized”. For clarity, the authors should provide a descriptive statistics table of all the CSA practices under study and the corresponding adopters and non-adopters.
4. GSEM's first mention in the body of text was not fully written. Authors should check and adjust the same for other acronyms. What are HYV and SWC? Please write their full meanings before continuing with Acronyms.

**Conclusion**

1. Check and recast: “The result from the PSM showed that using all CSA practices drops the level of MPI 342 from 0.04-0.20.”

**Recommendation**

1. The manuscript states that 2SLS is used to address endogeneity between CSA adoption and multidimensional poverty, yet the conclusion recommends that future studies should account for endogeneity and feedback loops between technology adoption and poverty outcomes. This appears inconsistent and requires clarification. Do the authors consider the current 2SLS approach sufficient, or does it only partially address the endogeneity concern?

.
