# Supplementary material for: Multidimensional poverty reduction effect of climate smart agriculture practices among rural households in Siltie Zone, Central Ethiopia
Source: PLoS One. 2026 May 4;21(5):e0348583. doi: 10.1371/journal.pone.0348583 (PMC13138676; doi:10.1371/journal.pone.0348583)
Supplement: Attachment — Submitted filename: Response to Reviewers.docx [file pone.0348583.s003.docx]

**Response to Editor and Reviewers**

| **Comments and Suggestions** | **Author’s Response** |
| --- | --- |
| **Editor Comments** | |
| 1. Please ensure that your manuscript meets PLOS ONE's style requirements, including those for file naming. | Dear editor, Thanks for your valuable comment and we considered all the PLOS ONE's style requirements, including those for file naming. |
| 2. In the ethics statement in the Methods, you have specified that verbal consent was obtained. Please provide additional details regarding how this consent was documented and witnessed, and state whether this was approved by the IRB | Thank you again and we included a sentence ‘approved by the IRB’ |
| 3. Thank you for stating the following in the Competing Interests section:  The authors have declared that no competing interests exist. Confirm that this does not alter your adherence to all PLOS ONE policies on sharing data and materials, by including the following statement: "This does not alter our adherence to PLOS ONE policies on sharing data and materials.” Include in cover letter. | Thank you again and we included a sentence "This does not alter our adherence to PLOS ONE policies on sharing data and materials.” As your direction. |
| 4. Please update your Data Availability statement in the submission form accordingly. | Thank you, there is no restriction, The data used for this study is publicly available at  10.6084/m9.figshare.31889473 |
| 5. Please ensure that you refer to Figures 1, 2, 3, and 4 in your text as, if accepted, production will need this reference to link the reader to the figures. | Dear editor we have completed as your directions, |
| 6. Replace or remove figure 2 because of permission | I removed the figure |
|  |  |
| **Reviewers Comments** | |
| **Reviewer 2 Comments** | |
| 1. I think it is better to add a sentence or two what smart agriculture means (line 46) | We agree with the reviewer. A clear definition of Climate-Smart Agriculture (CSA) has been added to improve conceptual clarity for readers unfamiliar with the term.in page 3, line 46-48. |
| 2. Here it is expected an explanation why you prefer MPI effects (line 55) | We appreciate this important suggestion. The manuscript has been revised to justify the use of the Multidimensional Poverty Index (MPI) instead of income-based poverty measures on page 3, line 56-59. |
| 3. You better detail factors in conceptual framework to make it more clearly to your reader. | We have revised the conceptual framework by explicitly detailing variables under each category to enhance clarity on page 4, line 67. |
| 4. “They utilize MPI and also panel data… what makes your approach outsmart needs explanation.” Line 100-102 | We thank the reviewer for this insightful observation. We have clarified the contribution of this study relative to existing literature. While previous studies often rely on panel data to analyze the relationship between agricultural practices and poverty, this study employs a combination of Propensity Score Matching (PSM), Two-Stage Least Squares (2SLS), and Generalized Structural Equation Modeling (GSEM). This integrated approach allows us to simultaneously address selection bias, control for endogeneity, and examine structural pathways through which CSA adoption affects multidimensional poverty. |
| 5. Permission for map | I have removed it from the manuscript because unable to get the required permission. |
| 6. Sampling Technique Justification | We have expanded the methodology section to clearly justify the sampling approach used. |
| 7. I feel three variables need reconsider… is all non-adopters female?” | We carefully re-examined and corrected inconsistencies in the descriptive statistics |
| 8. income interpretation is incorrect, it is a continuous variable | We appreciate this correction and have revised the interpretation accordingly. Refer page 29, line 364-367 |
| 9. It is recommended first to estimate impact via PSM, then 2SLS, finally GSEM | We agree and have reorganized the empirical analysis accordingly. |
| 10. Reconsider the interpretation in page 24, line 308-309—is that in percent? | Dear reviewer thank you for the right comment, We have clarified the interpretation and explicitly changed the measurement units to percent |
| 11. Move the statement on line to the recommendation section. | Right comment, We agree and have moved the statement to the recommendation section. |
| 12. statement on page 28, line 352 lacks meaning | The sentence has been rewritten for clarity and coherence |
| **Reviewer 1 Comments** | |
| 13. What is Kebele? What is climate risk? | Thank you for this important comment. We have clarified these concepts in the methodology section page 7, line 129 |
| 14. Justify why GSEM, PSM, and 2SLS are appropriate | We expanded the justification and clearly defined the analytical role of each model. Refer page 8-9, line 155-166 |
| 15. Clarify endogenous and exogenous variables. | We now clearly list all variables in the model. Refer page 9, line 171-178 |
| 16. clearly list descriptive statistics table of CSA practices | Dear reviewers we appreciated for this valuable comment and we do as your direction on page 11-12, line 231-235 |
| 17. What are the roles of the three models? Are they for robustness or address different econometric concerns? | We clarified that the models address different econometric issues. Refer on page 8-9, line 155-166 |
| 18. Clarify MPI construction. | Thank you for the right concern and We expanded the MPI explanation. Look page 8, line 149-154 |
| 19. Give theoretical justification for instrumental variables used | Thank you for the great point rose. I put the justification on page 9, line 171-178 |
| 20. Explain ATT preference. | Explained with reasoning. Refer on page 11, line 211-214 |
| 21. What are the implications that most households were middle age? | Thank you explained well. Look page 12, line 246-248 |
| 22. correct the interpretation the effect of CSA adoption on MPI on conclusion section | We appreciated your right feedback and follow the direction suggested. Page 29, line 379-380 |
