# Supplementary material for: Multidimensional poverty reduction effect of climate smart agriculture practices among rural households in Siltie Zone, Central Ethiopia
Source: PLoS One. 2026 May 4;21(5):e0348583. doi: 10.1371/journal.pone.0348583 (PMC13138676; doi:10.1371/journal.pone.0348583)
Supplement: Attachment — Submitted filename: Comments to authors_Plosone_review.docx [file pone.0348583.s004.docx]

**Comments to authors**

While I acknowledge the authors’ efforts to revise the manuscript and respond to earlier comments, a few important concerns persist. Addressing these issues would further enhance the rigor and overall quality of the study.

1. Authors should cite necessary references to support this claim - “Each indicator was weighted following standard 143 multidimensional poverty measurement procedures and with cut-off 0.33.” since it is a standard.
2. Line 302, “as tables 8 show…” recast to “Table 8 shows”
3. “The interpretation of the Rosenbaum sensitivity analysis appears to be inaccurate. Specifically, the statement that “the two groups should have differed by up to 780%” is not a correct representation of the Γ (gamma) parameter. In the Rosenbaum bounds framework, Γ reflects the extent to which unobserved factors could influence the odds of treatment assignment, not a percentage difference between groups.

The authors are advised to carefully cross-check this interpretation and revise the explanation in line with standard econometric literature. A more appropriate interpretation would express Γ in terms of how many times more likely one unit could receive treatment relative to another due to unobserved heterogeneity. Line 315

4. Bayesian information criterion - add the acronym once before subsequent mention on line 336
5. From Line 141 to 156, authors should provide relevant references. Same as 162 to 169
