# Supplementary material for: Multidimensional poverty reduction effect of climate smart agriculture practices among rural households in Siltie Zone, Central Ethiopia
Source: PLoS One. 2026 May 4;21(5):e0348583. doi: 10.1371/journal.pone.0348583 (PMC13138676; doi:10.1371/journal.pone.0348583)
Supplement: Attachment — Submitted filename: Response_to_Reviewers_auresp_2.docx [file pone.0348583.s005.docx]

**Response to Editor and Reviewers**

| **Comments and Suggestions** | **Author’s Response** | |
| --- | --- | --- |
| **Reviewer Comments** | | |
| 1. Authors should cite necessary references to support this claim - “Each indicator was weighted following standard MPI measurement procedures and with cut-off 0.33.” since it is a standard. (line 142-143) | | We agreed with the reviewer. And added the necessary references. Find on page 8, line 142. |
| 2. Line 302, “as tables 8 show…” recast to “Table 8 shows” | | We thank the reviewer for this insightful observation. We made correction as directed on page 24, line 304. |
| 3. “The interpretation of the Rosenbaum sensitivity analysis appears to be inaccurate. Specifically, the statement that “the two groups should have differed by up to 780%” is not a correct representation of the Γ (gamma) parameter. In the Rosenbaum bounds framework, Γ reflects the extent to which unobserved factors could influence the odds of treatment assignment, not a percentage difference between groups.(Line 315) | | We have revised the interpretation based on reviewers comment and direction. Refer on page 4, line (315-320). |
| 4. Bayesian information criterion - add the acronym once before subsequent mention (line 336) | | We thank the reviewer for this insightful observation. We added the acronyms with explanation on the first place. On page 29, line (338-339). |
| 5. From Line 141 to 156, authors should provide relevant references. Same as 162 to 169 | | We agreed with the reviewers comment and added the necessary references. Find on page 9, line 154-156, and on line 159,161-162 and 167. |
